# Supplementary material for: Anti-biofilm Fe3O4@C18-[1,3,4]thiadiazolo[3,2-a]pyrimidin-4-ium-2-thiolate Derivative Core-shell Nanocoatings
Source: Materials (Basel). 2020 Oct 17;13(20):4640. doi: 10.3390/ma13204640 (PMC7603173; doi:10.3390/ma13204640)
Supplement: Supplementary file 1 [file materials-13-04640-s001.pdf]

materials

Supplementary Materials

Anti-biofilm Fe_3O_4@C_{18}-[1,3,4]thiadiazolo[3,2-a]pyrimidin-4-ium-2-thiolate Derivative Core-shell Nanocoatings

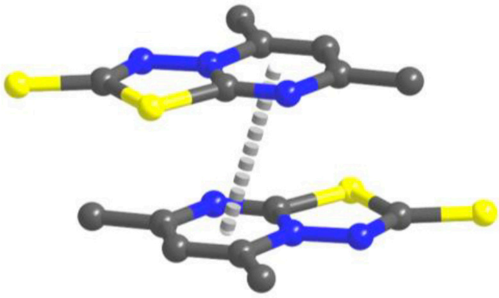

Figure S1. Supramolecular dimer of (**1**) in the solid state.

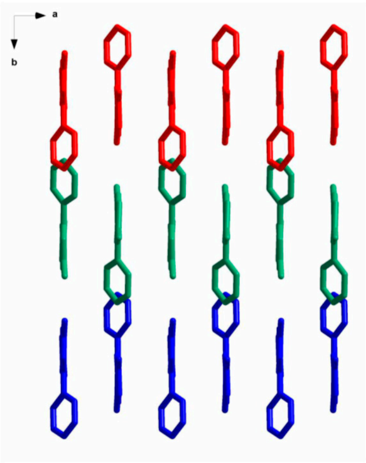

Figure S2. Crystal packing of (**2**) in the *ab* plane.

Publisher's Note: MDPI stays neutral with regard to jurisdictional claims in published maps and institutional affiliations.

© 2020 by the authors. Submitted for possible open access publication under the terms and conditions of the Creative Commons Attribution (CC BY) license (http://creativecommons.org/licenses/by/4.0/).
